# Machine learning and WGCNA reveal the PVT1/miR-143–3p/CDK1 ceRNA axis as a key regulator in NSCLC

**DOI:** 10.1016/j.bbrep.2025.102292

**Published:** 2025-09-30

**Authors:** Arash Safarzadeh, Setareh Ataei, Arezou Sayad, Soudeh Ghafouri-Fard

**Affiliations:** Department of Medical Genetics, Shahid Beheshti University of Medical Sciences, Tehran, Iran

**Keywords:** Non-small cell lung cancer, Machine learning, PVT1, miR-143–3p, CDK1

## Abstract

Machine learning has provided novel tools for analysis of multi-omics data for subgroups recognition in cancer to reach a clinically meaningful classification of cancer and identification of potential biomarkers. In this work, we retrieved mRNA, lncRNA, miRNA and protein expression data of non-small cell lung cancer (NSCLC) samples and used different machine learning methods for biomarker selection, diagnostic validation, construction of competing endogenous RNA network, identification of the hub axes and drug prediction. Integration of multi-omics data and machine learning resulted in identification of CDK1, TOP2A, AURKA, TPX2, BUB1B, and CENPF as key biomarkers in NSCLC. We also identified the PVT1/miR-143–3p/CDK1 axis and its associated transcription factors (FOXC1, YY1, and GATA2) as a potential regulatory network for additional investigations. These findings increase the understanding of NSCLC molecular processes and provide a foundation for developing targeted therapies and diagnostic tools.

## Highlights

• CDK1, TOP2A, AURKA, TPX2, BUB1B, and CENPF are key biomarkers in NSCLC.
• PVT1/miR-143–3p/CDK1 axis and its associated transcription factors are potential regulatory network.
• FOXC1, YY1, and GATA2 transcription factors are involved in the pathogenesis of NSCLC.

## 1 Introduction

Lung cancer is the second most diagnosed cancer among both sexes and is among those with the least favorable prognosis [1]. The low survival rate is largely due to the first-line therapy's inefficacy, stemming from insufficient knowledge about molecular-level tumor heterogeneity [2,3]. This cancer is categorized to two main classes, namely non-small cell lung cancer (NSCLC) and SCLC with the former accounting for 85 % of cases [4]. Integrative molecular characterization researches have led to the identification of novel molecular signatures in the lung cancer and its distinct subtypes at genomic level, DNA methylation patterns, as well as mRNA expression, miRNA expression, and protein expression levels [4]. These efforts have been directed towards establishment of clinically meaningful tumor classification as well as a better identification of the molecular features of lung cancer.

The limited sensitivity of current imaging techniques makes it difficult to detect NSCLC early, and acquired resistance to immunotherapy and targeted therapies frequently compromises the effectiveness of treatment [5,6].

Recent advance in the high-throughput sequencing techniques has produced huge amounts of molecular data at various omic levels, facilitating understanding of the molecular mechanisms of disease heterogeneity. Meanwhile, machine learning has provided novel tools for assessment of multi-omics data for molecular profiling in lung cancer [7].

Although many molecular alterations in NSCLC have been studied individually, the complex interactions among coding and non-coding RNAs, as well as their regulatory networks, are not fully elucidated. Integrative multi-omics analyses combined with machine learning provide a promising approach to uncover novel biomarkers and regulatory axes that may improve diagnosis and therapy [8,9].

In this work, in addition to mRNA, lncRNA and miRNA data, we added protein expression data and used different machine learning methods for biomarker selection, diagnostic validation, construction of competing endogenous RNA (ceRNA) network, identification of the hub axes and drug prediction.

## 2 Materials and methods

**Fig. 1:**
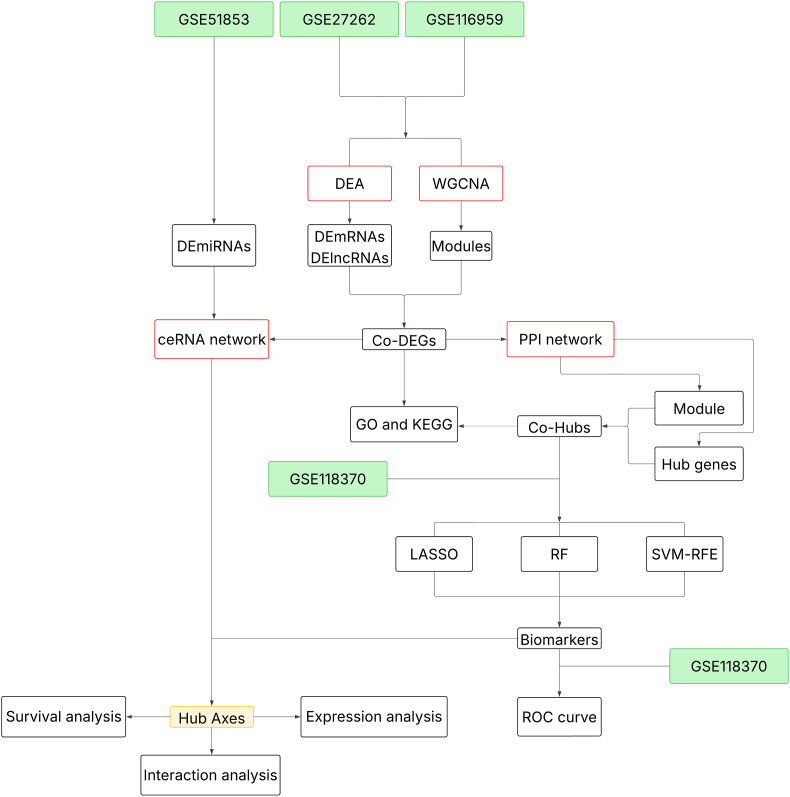
The study flowchart.

Fig. 1 shows an overview of the analytical workflow utilized in this study.

### 2.1 Data source

The datasets utilized were obtained from the Gene Expression Omnibus (GEO) database (https://www.ncbi.nlm.nih.gov/geo/) [10]. Specifically, GSE27262 ([HG-U133_Plus_2] Affymetrix Human Genome U133 Plus 2.0 Array; GPL570), GSE116959 (Agilent-039494 SurePrint G3 Human GE v2 8 × 60K Microarray 039381 [Probe Name version]; GPL17077), and GSE51853 (Agilent-015508 Human miRNA Microarray, pre-commercial version 6.0 [Unique miRNA version]; GPL7341) were selected for the identification of differentially expressed genes (DEGs). Additionally, GSE75037 (Illumina HumanWG-6 v3.0 Expression BeadChip; GPL6884) was employed for machine learning-based analysis, and GSE118370 ([HG-U133_Plus_2] Affymetrix Human Genome U133 Plus 2.0 Array; GPL570) was used to validate the identified biomarkers. Table 1 summarizes the essential characteristics of the included datasets.

**Table 1:** The information on the datasets from GEO.

Series	Platform	Samples	Component	Status
GSE27262	GPL570	25 Normal	mRNAs lncRNAs	Public on Apr 10, 2013
		25 Tumor		
GSE116959	GPL17077	11 Normal		Public on Sep 04, 2019
		57 Tumor		
GSE118370	GPL570	6 Normal		Public on Jan 23, 2019
		6 Tumor		
GSE51853	GPL7341	5 Normal	miRNAs	Public on Jun 19, 2014
		126 Tumor		
GSE75037	GPL6884	83 Normal	mRNAs lncRNAs	Public on Jun 01, 2016
		83 Tumor		

### 2.2 Identification of differentially expressed genes (DEGs)

To find differentially expressed mRNAs (DEmRNAs) and long non-coding RNAs (DElncRNAs), we analyzed the GSE27262 and GSE116959 datasets. Differentially expressed miRNAs (DEmiRNAs) were derived from the GSE51853 dataset. First, Gene expression matrices were initially normalized using the preprocessCore package (version 1.70.0). Then, All analyses were performed using the “limma” package (version 3.58.1) [11] in the R programming environment (version 4.3.2). A stringent cutoff was applied to define differential expression: for DEmRNAs and DElncRNAs, we used |log2 fold change| ≥ 1 with an adjusted P-value <0.05, while for DEmiRNAs, the threshold was set at |log2 fold change| ≥ 0.8 and adjusted P-value <0.05. A slightly reduced threshold was utilized for miRNAs, as they typically exhibit smaller fold changes relative to mRNAs; employing the same cutoff might omit biologically significant candidates. Principal component analysis (PCA) by prcomp function was conducted to assess sample distribution, and volcano plots were generated to visualize the differentially expressed genes using “EnhancedVolcano” package (version 1.10.0) [12]. A Venn diagram using InteractiVenn [13] was also constructed to illustrate the overlap of DEGs between the datasets.

### 2.3 Identification of disease-related shared genes

Weighted Gene Co-expression Network Analysis (WGCNA) was performed on the GSE27262 and GSE116959 datasets to identify biologically meaningful transcriptome co-expression modules. Initially, hierarchical clustering was conducted to evaluate sample quality within each dataset. Subsequently, the pickSoftThreshold function in the “WGCNA” package (version 1.72.5) [14] was used to determine the optimal soft-thresholding power (β), ensuring the construction of a scale-free network topology. Genes exhibiting similar expression profiles were grouped into distinct modules, each assigned a unique color, with a minimum module size of 30 genes and a merge cut height of 0.25. Modules showing the strongest correlation with clinical traits or biological conditions were identified as key modules in each dataset. The intersecting gene sets from the key modules of both datasets were then extracted to determine shared genes. Finally, these overlapping genes were further intersected with the previously identified DEGs to pinpoint the most functionally relevant candidates.

### 2.4 Gene enrichment analysis

To investigate the biological function of the shared genes, Gene Ontology (GO) [15,16] enrichment analysis and Kyoto Encyclopedia of Genes and Genomes (KEGG) [17] pathway analysis were accomplished using R. The GO analysis encompassed three major categories: biological process (BP), cellular component (CC), and molecular function (MF). KEGG analysis was conducted to explore gene-associated metabolic and signaling pathways. Functional enrichment and data visualization were carried out using the clusterProfiler package (version 4.10.0) [18] in conjunction with ggplot2 (version 3.5.1) [19]. An adjusted P-value threshold of <0.05 was applied to define statistically significant results. The top 10 enriched terms were presented as bar plots and dot plots.

### 2.5 Construction of the Protein–Protein interaction (PPI) network and recognition of hub genes

The intersected gene set was uploaded to the STRING database (https://string-db.org/) [20] to construct a PPI network, with the medium confidence score >0.4 and medium DFR stringency <0.05. The resultant network was then imported into Cytoscape software (version 3.10.3) [21] for improved visualization and further analysis. To identify key regulatory genes, the CytoHubba plug-in (version 0.1) [22] was used to calculate multiple topological parameters, including Maximal Clique Centrality (MCC), Degree, Betweenness, Closeness, Stress, and Radiality. A composite scoring system was developed based on these metrics, and the top 10 genes with the highest overall scores were selected as hub genes. In parallel, the MCODE plug-in (version 2.0.3) [23] was applied to detect densely connected submodules within the PPI network, and the module with the highest score was retained. Finally, the overlapping genes between the top 10 hub genes and the genes from the highest-scoring MCODE module were identified for subsequent analysis. Also, GO and KEGG pathway enrichment of the overlapping genes were performed using ClueGO plug-in (version 2.5.10) [24].

### 2.6 Machine learning-based biomarker selection and diagnostic validation in the test dataset

To further identify key biomarkers from the set of overlapping genes, we employed three machine learning algorithms—Least Absolute Shrinkage and Selection Operator (LASSO), Random Forest (RF), and Support Vector Machine-Recursive Feature Elimination (SVM-RFE)—using the GSE75037 dataset. The LASSO model was executed via the glmnet package (version 4.1.8) [25], the RF model using the randomForest package (version 4.7.1.1) [26], and the SVM-RFE approach was applied through the e1071 package (version 1.7.14) [27]. The genes identified by each method were compared using a venn diagram, and the overlapping candidates were selected as the most robust biomarkers. These selected genes were then evaluated in the validation dataset GSE118370. Diagnostic performance was evaluated using receiver operating characteristic (ROC) curve construction in GraphPad Prism version 9.0.0 for Windows, and the area under the curve (AUC) was calculated to quantify diagnostic accuracy.

### 2.7 Construction of CeRNA network, identification of the hub axes and drug prediction

To make the ceRNA network, we first retrieved interactions between DElncRNAs and their target miRNAs using the miRcode [28] and DIANA-LncBase v3 [29] databases. Interactions involving miRNAs that were not present in the list of DEmiRNAs were excluded. Next, we identified the interactions between DEmiRNAs and their target mRNAs using the miRDB [30] and TargetScan 8.0 [31] databases. Similarly, interactions involving mRNAs that were not among the DEmRNAs were removed. We further filtered the interactions to retain only those in which the expression pattern of the DElncRNA was inverse to that of the DEmiRNA, and the expression of the DEmiRNA was inverse to that of the DEmRNA. Finally, the resulting ceRNA network was visualized using Cytoscape. To identify hub regulatory axes, we searched for the machine learning–identified biomarkers within the constructed ceRNA network. The axes containing these biomarkers were defined as hub axes. The ceRNA network and this axis were not constructed based on correlations between genes, but solely based on interactions and reverse expression patterns obtained from databases; therefore, this axis is considered hypothetical. Also, Using NetworkAnalyst 3.0 [32] and JASPAR [33] databases, transcription factors (TFs) that regulate the biomarkers were identified. Using the NcPath database [34], we investigated the KEGG pathways associated with the identified hub axis. To explore potential therapeutic agents for NSCLC, the identified biomarkers were screened using the Drug-Gene Interaction Database (DGIdb) [35]. Candidate drugs predicted to target these biomarkers were collected, and their interactions were subsequently visualized through a biomarker–drug interaction network constructed using Cytoscape software.

### 2.8 Validation of ceRNA hub axis using external databases

The genes within the hub axis were assessed using the UALCAN database [36] to assess their expression in the TCGA-LUAD dataset, and the Kaplan–Meier Plotter database (https://kmplot.com/analysis/) was employed to evaluate their association with overall survival in the same dataset. Additionally, interactions between the lncRNA and miRNA, as well as between the miRNA and mRNA, were investigated using the starBase v2.0 database [37] based on Ago CLIP-seq data and experimental findings.

## 3 Results

### 3.1 Differential expression analysis (DEA)

**Fig. 2:**
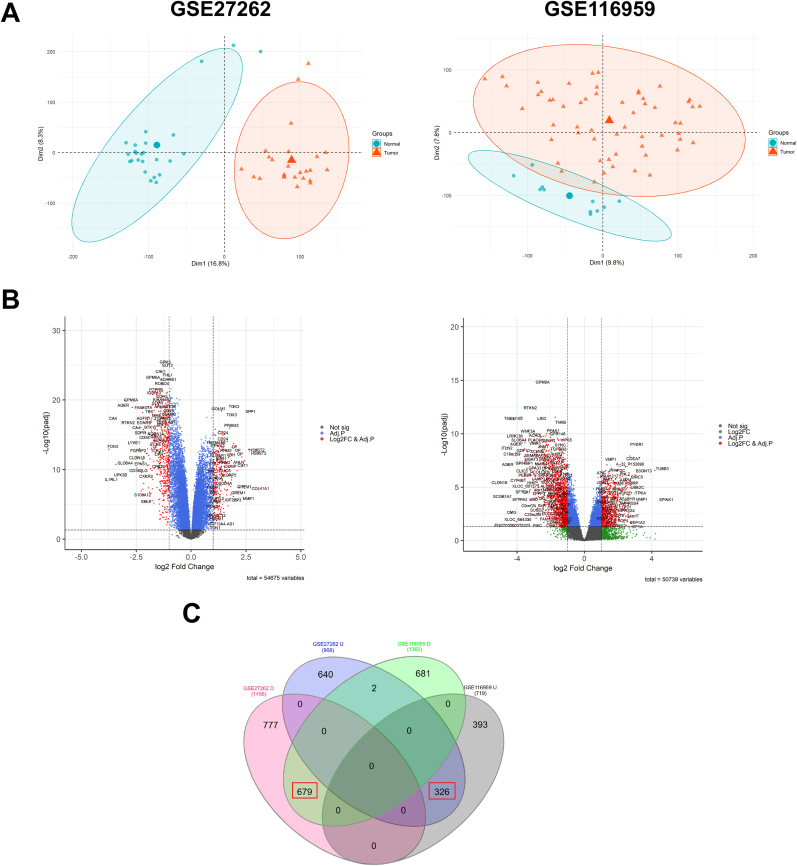
**Detection of differentially expressed genes.** (A) Principal component analysis (PCA) plot of datasets GSE27262 and GSE116959. (B) Volcano plots displaying the distribution of DEGs identified in GSE27262 and GSE116959. (C) Venn diagram illustrating the overlapping DEGs shared between the two datasets.

Differential expression analysis of the GSE27262 and GSE116959 datasets identified 2424 DEGs (comprising 968 upregulated and 1456 downregulated genes) and 2081 DEGs (719 upregulated and 1362 downregulated genes), respectively. Additionally, analysis of GSE51853 revealed 21 differentially expressed miRNAs (DEmiRNAs). PCA was conducted to find distribution of samples, and volcano plots were used to demonstrate the significantly altered genes (Fig. 2A and B). The intersection between the DEGs of dataset GSE27262 and those of dataset GSE116959 identified 326 commonly upregulated and 679 commonly downregulated DEGs (Fig. 2C).

### 3.2 Weighted gene Co-expression network analysis (WGCNA)

**Fig. 3:**
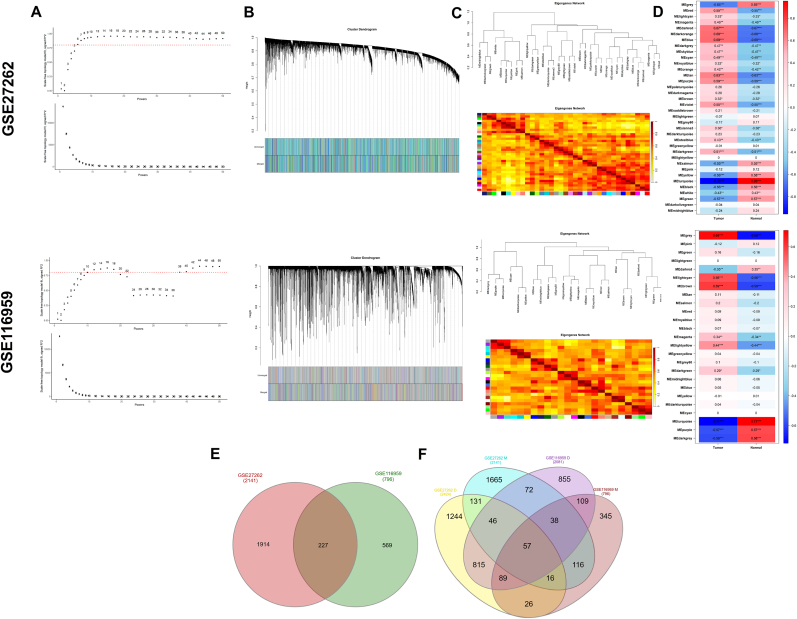
**Weighted gene co-expression network analysis and key modules identification**. (A) Network topology analysis for selecting the optimal soft-thresholding power (β) in datasets GSE27262 and GSE116959. (B) Dendrograms of gene clustering and corresponding co-expression modules identified by WGCNA in GSE27262 and GSE116959, with each module represented by a distinct color. (C) Heatmap displaying module correspondence between GSE27262 and GSE116959. (D) Heatmap illustrating the correlation between clinical traits and gene modules in both datasets. (E) Venn diagram indicating the overlap between module genes from GSE27262 and GSE116959. (F) Venn diagram showing the intersection between the intersected DEGs and the overlapping module genes.

WGCNA was used to identify key gene modules associated with NSCLC. The optimal soft-thresholding power (β) was determined to be 6 for the GSE27262 dataset and 12 for GSE116959 (Fig. 3A). In GSE27262, a total of 37 modules were detected, among which the red, darkred, darkorange, blue, tan, purple, violet, and darkgreen modules exhibited the strongest positive correlations with clinical traits. Similarly, analysis of GSE116959 revealed 25 modules, with the lightcyan and brown modules showing the highest positive correlations (correlation coefficient >0.5; Fig. 3B–D). A Venn diagram analysis identified 227 common genes from the positively correlated modules across both datasets (Fig. 3E). Subsequently, these 227 overlapping genes were intersected with 1005 intersected DEGs, leading to the identification of 57 shared genes relevant to NSCLC (Fig. 3F).

### 3.3 Functional enrichment analysis

**Fig. 4:**
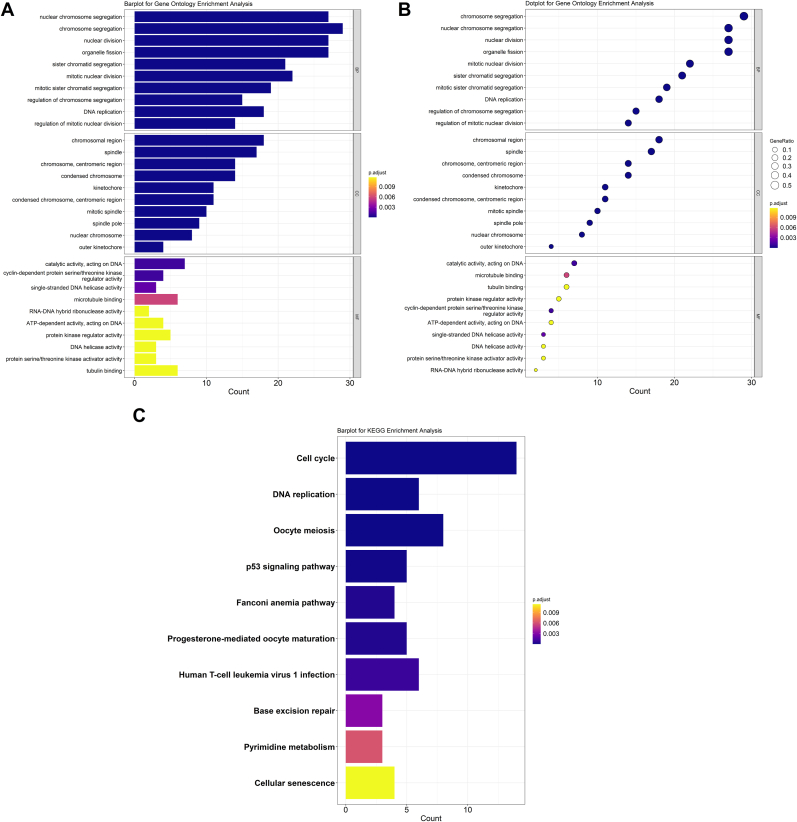
**Shared genes function enrichment analysis. (A)** Bar plot illustrating the top 10 Gene Ontology (GO) terms significantly enriched among the shared genes. **(B)** Dot plot representing the top 10 significantly enriched GO terms. **(C)** KEGG pathway analysis showing the top 10 enriched signaling pathways.

GO and KEGG enrichment analyses were conducted on the 57 identified shared genes. A total of 280 significantly enriched GO terms were obtained, comprising 217 biological processes (BP), 37 cellular components (CC), and 26 molecular functions (MF). The BP terms were primarily associated with “nuclear chromosome segregation,” “chromosome segregation,” and “nuclear division.” Enriched CC terms included “chromosomal region,” “spindle,” and “chromosome, centromeric region.” The MF category was mainly enriched in “catalytic activity acting on DNA,” “cyclin-dependent protein serine/threonine kinase regulator activity,” and “single-stranded DNA helicase activity” (Fig. 4A and B). KEGG analysis revealed 11 significantly enriched pathways, predominantly related to the “cell cycle,” “DNA replication,” and “oocyte meiosis” (Fig. 4C). Collectively, these findings indicate that NSCLC is closely linked to biological processes involved in “cell growth and death” as well as “replication and repair."

### 3.4 PPI network construction and identify the hub genes

**Fig. 5:**
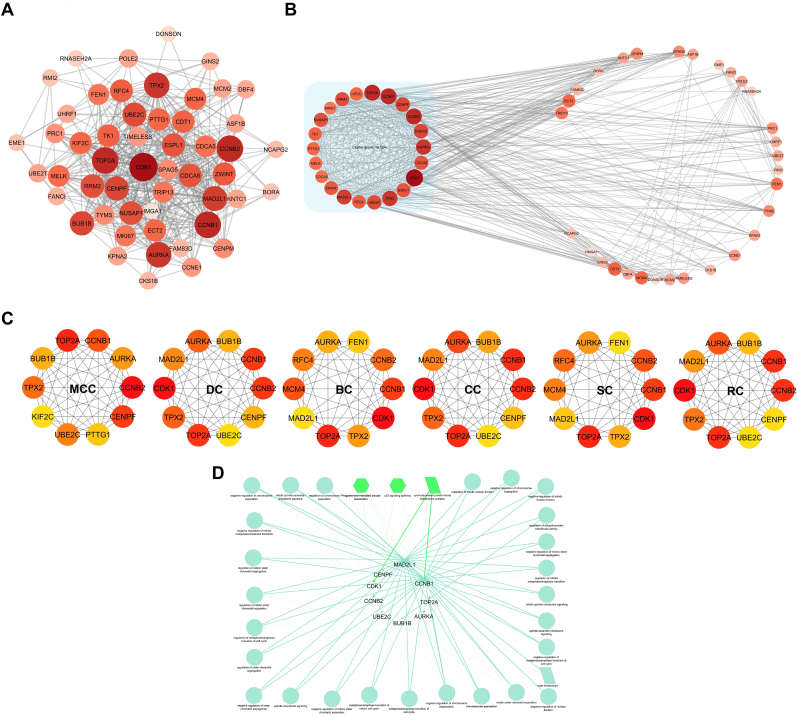
**Analysis of the protein-protein interaction (PPI) network and identification of hub genes.** (A) Visualization of the PPI network. Node color intensity, ranging from white to red, reflects increasing betweenness centrality, while node size corresponds to the degree of connectivity. (B) The highest-scoring PPI module extracted from the network. (C) Top 10 hub genes identified based on multiple centrality algorithms, including MCC, DC, BC, CC, SC, and RC. (D) Gene Ontology (GO) and KEGG pathway enrichment analysis of the hub genes represented as a functional interaction network.

PPI network was constructed for the 57 shared genes, with a total of 57 nodes and a total of 478 interactions between nodes (Fig. 5A). The key PPI network modules were found using the MCODE plugin. The following thresholds were used for the analysis: Max Depth = 100, K-core = 2, Degree Cutoff = 2, and Node Score Cutoff = 0.2. The most significant module, with a score of 19.524, included the following genes: TOP2A, CCNB1, CENPF, CCNB2, BUB1B, AURKA, CDCA8, CDK1, ESPL1, TPX2, UBE2C, RFC4, MAD2L1, ZWINT, CDCA5, MELK, PTTG1, TK1, NUSAP1, MKI67, RRM2, and KIF2C (Fig. 5B). Additionally, using the CytoHubba plugin, various centrality scores—including MCC, degree centrality, betweenness centrality, closeness centrality, stress centrality, and radiality centrality—were calculated for all genes (Fig. 5C). After normalizing the scores, the values for each gene were multiplied to generate a combined score (Table S1). The top 10 genes with the highest combined scores were identified: CDK1, TOP2A, CCNB1, CCNB2, AURKA, TPX2, MAD2L1, BUB1B, UBE2C, and CENPF. All of these genes were also present in the identified module; additionally, GO and KEGG pathway enrichment analyses were conducted for these genes using the ClueGO plugin (Fig. 5D).

### 3.5 Identification of core genes via machine learning algorithms and validation of them

**Fig. 6:**
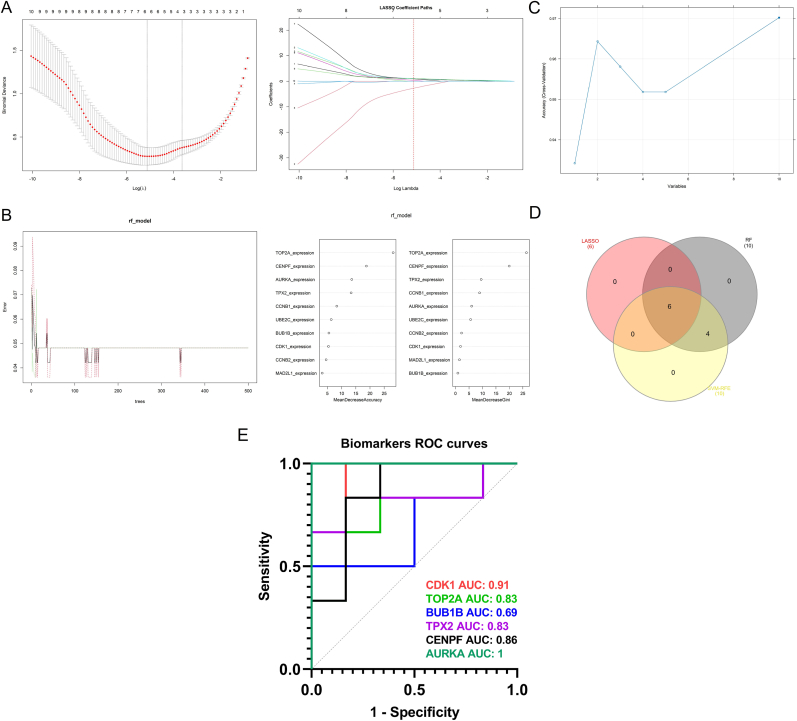
**Machine learning-based identification and validation of final candidate biomarkers. (A)** Six genes were selected using the LASSO regression model based on the minimum binomial deviance. **(B)** Ten important genes were identified using the Random Forest algorithm based on their feature importance scores. **(C)** The Support Vector Machine-Recursive Feature Elimination method also selected ten genes with the highest importance scores. **(D)** A Venn diagram was used to determine six overlapping genes shared across the three algorithms. **(E)** Receiver Operating Characteristic (ROC) curve analysis of the six consensus genes was conducted using the GSE118370 validation dataset.

To improve the selection of hub genes identified in earlier analyses, three machine learning methods were applied: LASSO, RF, and SVM-RFE. LASSO regression identified six genes—AURKA, CDK1, TPX2, TOP2A, BUB1B, and CENPF—as potential markers (Fig. 6A). The RF model recognized all ten previously determined hub genes (Fig. 6B), and similarly, the SVM-RFE algorithm also selected the same ten genes (Fig. 6C). By intersecting the results from all three models using a Venn diagram, six genes (AURKA, CDK1, TPX2, TOP2A, BUB1B, and CENPF) were confirmed as key candidates (Fig. 6D). Further validation using the external dataset GSE118370 showed that CDK1, TOP2A, TPX2, CENPF, and AURKA exhibited strong diagnostic capabilities for NSCLC, with each gene achieving an AUC greater than 0.8 (Fig. 6E).

### 3.6 ceRNA and gene-drug network construction

**Fig. 7:**
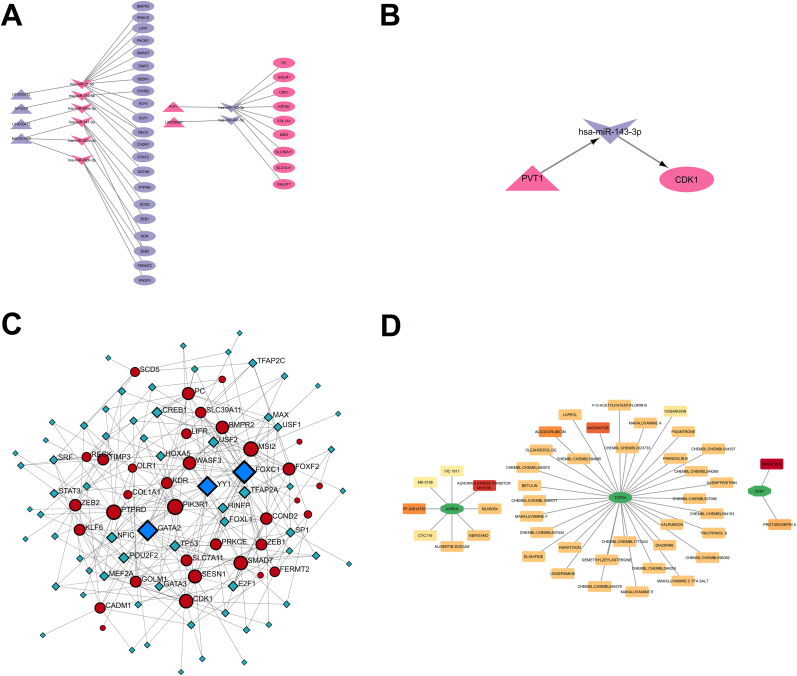
**Visualization of the constructed regulatory networks. (A)** The ceRNA (lncRNA–miRNA–mRNA) network: pink nodes represent upregulated genes, while purple nodes denote downregulated genes. **(B)** Central regulatory axis within the ceRNA network. **(C)** TF–mRNA interaction network: green nodes indicate TFs, dark red nodes represent mRNAs, and blue nodes highlight the top three TFs with the highest degree of connectivity. **(D)** Gene–drug interaction network: the intensity of the red color reflects the interaction score between each gene and drug, with darker shades indicating stronger interactions.

Based on the miRcode and lncBase databases, it was identified that 10 miRNAs interact with 6 lncRNAs. These interactions were selected according to opposing expression patterns, and the corresponding genes were also present in the list of differentially expressed genes (DEGs). Furthermore, using the miRDB and TargetScan databases, it was found that 30 mRNAs interact with the aforementioned 10 miRNAs. These interactions were also selected based on inverse expression patterns, and the involved mRNAs were included in the DEG list as well. Finally, the ceRNA network was constructed using Cytoscape software (Fig. 7A). To identify a hub axis, the biomarkers obtained from machine learning were searched within the ceRNA network. Among these biomarkers, only CDK1 was present in the network. Therefore, the interactions involving CDK1 were considered as a key axis, identified as the PVT1/hsa-miR-143–3p/CDK1 axis (Fig. 7B). Subsequently, using the NetworkAnalyst and JASPAR databases, transcription factors (TFs) associated with the mRNAs in the ceRNA network were analyzed. The results indicated that FOXC1, YY1, and GATA2—with the highest degree values—are the main transcriptional regulators of CDK1 (Fig. 7C). KEGG pathway enrichment analysis of the hub axis genes using the NcPath database revealed that this hub axis is most significantly involved in the Endocrine resistance pathway, showing the lowest P-value among enriched pathways (Table S2). Also, analysis using the Drug–Gene Interaction Database (DGIdb) identified a total of 225 potential therapeutic compounds associated with the selected biomarkers (Table S3). The resulting drug–biomarker interaction network revealed that 50 compounds were predicted to target CDK1, including Aruncin B, Protuboxepin A, and Dinaciclib. Additionally, 58 compounds were found to interact with AURKA, such as the Aurora A kinase inhibitor MK5108, PF-03814735, and Alisertib sodium. For TOP2A, 117 candidate compounds were identified, including Amonafide, Aldoxorubicin, and CHEMBL:CHEMBL596082. In contrast, no drugs were predicted to target either CENPF or TPX2. We selected the interactions with an interaction score >0.5 for visualization in Cytoscape, as this threshold represents a moderate to high confidence in drug–target associations (Fig. 7D).

### 3.7 Validation of the hub axis

**Fig. 8:**
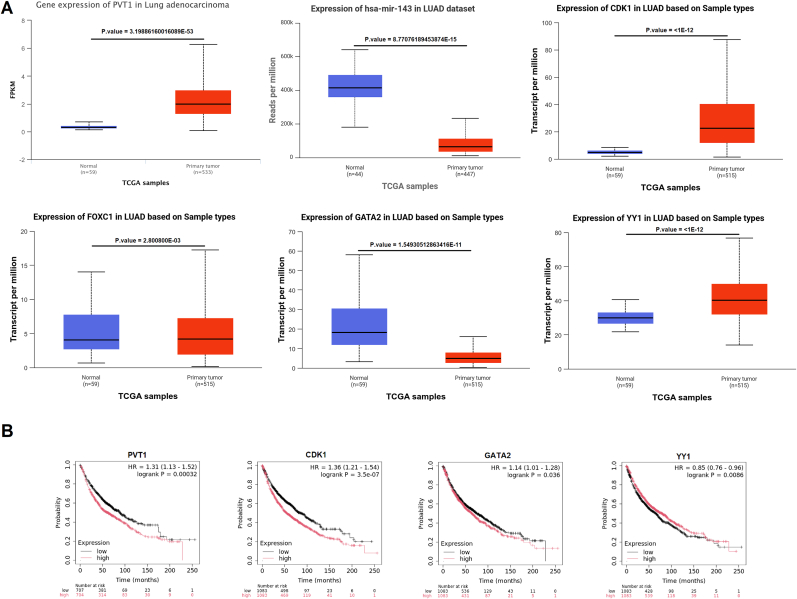
**Validation of the ceRNA hub axis. (A)** The expression of the genes in TCGA-LUAD samples. **(B)** The overall survival of the genes in TCGA-LUAD samples.

According to the UALCAN database, the expression levels of PVT1, hsa-miR-143–3p, CDK1, FOXC1, YY1, and GATA2 show statistically significant differences between primary tumor samples and normal samples in the TCGA-LUAD dataset (Fig. 8A). Additionally, based on the Kaplan–Meier Plotter database, CDK1, PVT1, GATA2, and YY1 exhibit overall survival (OS) p-values less than 0.05 (Fig. 8B). The interaction between PVT1 and hsa-miR-143–3p was characterized as a 7mer-m8 site type with a TDMDScore of 0.9593, indicating a strong potential for target-directed miRNA degradation. This interaction was supported by one Argonaute CLIP-seq experiment (AgoExpNum = 1) without any observed cleavage events (CleaveExpNum = 0). The phyloP score of −0.101 indicates limited evolutionary conservation, while the interaction was detected across eight cancer types in TCGA, suggesting pan-cancer relevance. Similarly, the interaction between hsa-miR-143–3p and CDK1 showed a TDMDScore of 1.0546, also supported by one Ago CLIP-seq experiment and no cleavage events, with a phyloP score of 0.391 indicating moderate conservation. This interaction was observed in seven TCGA cancer types, highlighting its potential functional significance (Table S4).

## 4 Discussion

In this study, we used an inclusive bioinformatics approach along with machine learning to find key molecular signatures and regulatory networks in NSCLC. Our findings revealed critical genes, pathways, and interactions that may function as potential diagnostic biomarkers and therapeutic targets. Differential expression analysis of the GSE27262 and GSE116959 datasets identified thousands of DEGs, with 326 commonly upregulated and 679 downregulated genes, suggesting a strong molecular basis for NSCLC progression. WGCNA further refined these findings by identifying co-expressed gene modules. The intersection of DEGs and co-expression modules yielded 57 shared genes, reinforcing their potential role in NSCLC pathogenesis.

GO and KEGG enrichment analyses showed that the 57 shared genes are mainly implicated in cell cycle regulation, DNA replication, and chromosomal segregation. These findings align with the known hallmarks of cancer, particularly uncontrolled proliferation and genomic instability [38]. The strong association with mitotic processes underscores the potential of these genes as therapeutic targets, given their critical role in tumor growth.

PPI network analysis and machine learning approaches (LASSO, RF, and SVM-RFE) recognized six core genes—AURKA, CDK1, TPX2, TOP2A, BUB1B, and CENPF—as important biomarkers. These genes have central roles in cell cycle regulation, with CDK1, TOP2A, and AURKA representing particularly high diagnostic accuracy in external validation. Their consistent selection across multiple algorithms reinforces their reliability as NSCLC biomarkers.

Most notably, the constructed ceRNA network highlighted the PVT1/hsa-miR-143–3p/CDK1 axis as a critical regulatory mechanism in NSCLC. miR-143–3p has been identified as a tumor suppressor in several cancers through regulation of several signaling pathways, including the PI3K/Akt, Wnt/β-catenin, AKT/STAT3 and Ras/Raf/MEK/ERK pathways [39]. Additionally, PVT1 has been recognized as an essential element in the complex landscape of cancer pathogenesis, especially in lung cancer [40].

It has been demonstrated that aberrant activation of CDK1, a key regulator of the G2/M transition, promotes uncontrolled proliferation and resistance to apoptosis in lung adenocarcinoma [41,42]. By downregulating EGFR in NSCLC, miR-143–3p, on the other hand, acts as a tumor suppressor and has been shown to prevent cell proliferation and invasive potential [43]. To promote oncogene expression and tumor progression, PVT1, a well-characterized oncogenic lncRNA, can sequester tumor-suppressive miRNAs like a molecular sponge [44,45]. Our results suggest that the PVT1/miR-143–3p/CDK1 axis may be a crucial mechanism whereby PVT1 inhibits miR-143–3p activity, resulting in CDK1 derepression and improved cell cycle progression in NSCLC. Our study is the first to combine PVT1 and miR-143–3p within a ceRNA axis that converges on CDK1, offering a systems-level viewpoint that unifies non-coding RNA regulation with a central cell cycle kinase, even though both have been previously connected to lung cancer.

From a therapeutic perspective, this axis may offer multiple points of intervention. Restoration of miR-143–3p activity using synthetic mimics or inhibition of PVT1 through antisense oligonucleotides could reduce CDK1 expression indirectly, while CDK1 itself remains a promising target for small-molecule inhibitors such as Dinaciclib. These strategies may exert synergistic effects, blocking both upstream regulatory elements and downstream cell cycle drivers. Thus, the PVT1/miR-143–3p/CDK1 axis provides a rationale for exploring combined ncRNA-based and kinase-targeted therapies in NSCLC.

The PVT1/hsa-miR-143–3p/CDK1 axis was further supported by transcription factor analysis, identifying FOXC1, YY1, and GATA2 as key regulators of CDK1. It is worth mentioning that FOXC1, YY1, and GATA2 have also been reported to be the key regulators of DEGs in another bioinformatics analysis in NSCLC [46]. The involvement of these TFs suggests a complex transcriptional control mechanism influencing NSCLC progression.

The discovery that CDK1, TOP2A, AURKA, TPX2, BUB1B, and CENPF are key cell-cycle regulators in NSCLC emphasizes the ongoing contribution of mitotic dysregulation to tumor growth. According to earlier research, these genes' overexpression is linked to NSCLC's poor prognosis, increased proliferation, and genomic instability [47,48]. These genes' integration into the PVT1/miR-143–3p/CDK1 ceRNA axis offers a systems-level viewpoint that links central cell-cycle machinery and non-coding RNA regulation, which hasn't been thoroughly explained in NSCLC before.

Targeting several nodes of this axis may have synergistic therapeutic effects. In line with the justification for multi-targeted approaches in the treatment of lung cancer, for example, combining CDK1 inhibitors with techniques to restore tumor-suppressive miR-143–3p or inhibit oncogenic PVT1 may suppress cell-cycle progression and increase sensitivity to currently available chemotherapies [49].

While many of the genes found in this study have been previously reported in NSCLC, such as CDK1, TOP2A, AURKA, TPX2, BUB1B, and CENPF, as well as PVT1 and miR-143–3p, our study offers new insights by combining multiple transcriptomic datasets, co-expression network analysis, and machine learning approaches. We discovered the PVT1/miR-143–3p/CDK1 axis within a ceRNA network, in contrast to earlier research that looked at these molecules separately, suggesting that it may be a key regulatory mechanism in the development of NSCLC. Additionally, using transcription factor analysis, we identified FOXC1, YY1, and GATA2 as upstream regulators. Then, we combined this information with drug-gene interaction analysis to suggest possible therapeutic approaches. This integrative approach allows a more system-level understanding of NSCLC molecular regulation, offering hypotheses for experimental validation that extend beyond confirming previously reported findings. However, This axis is hypothetical and requires further experimental validation in future studies.

Drug-gene interaction analysis revealed multiple FDA-approved and investigational compounds targeting CDK1, AURKA, and TOP2A, including Dinaciclib (CDK1 inhibitor), Alisertib (AURKA inhibitor), and Amonafide (TOP2A inhibitor). Dinaciclib has been previously shown to induce anaphase catastrophe in lung cancer cells through suppression of CDK1 and CDK2 [50]. Safety and activity of Alisertib has been assessed in patients with different types of solid tumors, including SCLC and NCSLC in a five-arm phase 2 study [51]. These findings provide a rationale for repurposing existing drugs or developing novel therapies targeting these hub genes.

Validation using TCGA-LUAD data verified dysregulation of the PVT1/miR-143–3p/CDK1 axis in NSCLC patients. Survival analysis further showed that CDK1, PVT1, GATA2, and YY1 are meaningfully associated with poor OS, reinforcing their prognostic value. The strong TDMDScore for PVT1/miR-143–3p and miR-143–3p/CDK1 interactions implies a functionally relevant regulatory relationship, possibly contributing to NSCLC pathogenesis.

## 5 Limitations and future directions

While this study provides robust evidence from *in silico* analyses, experimental validation (*in vitro* and *in vivo* studies) is needed to confirm the mechanistic roles of the identified genes and regulatory networks. Moreover, further clinical studies are necessary to assess the therapeutic efficacy of the predicted drug candidates.

## 6 Conclusions

Taken together, this study integrates multi-omics data and machine learning to identify CDK1, TOP2A, AURKA, TPX2, BUB1B, and CENPF as key biomarkers in NSCLC. The PVT1/miR-143–3p/CDK1 axis and its associated transcription factors (FOXC1, YY1, and GATA2) signify a potential regulatory network for additional investigations. These findings not only improve our understanding of NSCLC molecular processes but also provide a foundation for developing targeted therapies and diagnostic tools.

### Data availability statement

The datasets (GSE27262, GSE116959, GSE118370, GSE51853 and GSE75037) presented in this study were obtained from the GEO (https://www.ncbi.nlm.nih.gov/geo/) database.

## Ethics approval and consent to participate

Not applicable.

## Author contributions (CRediT)

Arash Safarzadeh: Conceptualization, Study Design, Bioinformatics Analysis, Data Curation, Visualization, Writing – Original Draft. Setareh Ataei: Data Curation, Visualization. Arezou Sayyad: Supervision. Soudeh Ghafourifard: Writing – Review & Editing. All authors have read and approved the final manuscript.

## Consent to publish declaration

Not applicable.

## Clinical trial number

Not applicable.

## Funding statement

This research did not receive any specific grant from funding agencies in the public, commercial, or not-for-profit sectors.

## Declaration of competing interest

Authors declare no conflicts of interests.

## Data Availability

No data was used for the research described in the article.
